# Ti-based robust MOFs in the combined photocatalytic degradation of emerging organic contaminants

**DOI:** 10.1038/s41598-022-18590-1

**Published:** 2022-08-25

**Authors:** Sara Rojas, Jessica García-González, Pablo Salcedo-Abraira, Irene Rincón, Javier Castells-Gil, Natalia M. Padial, Carlos Marti-Gastaldo, Patricia Horcajada

**Affiliations:** 1 Advanced Porous Materials Unit (APMU), IMDEA Energy Institute. Av. Ramón de La Sagra 3, 28935 Móstoles-Madrid, Spain; 2 Department of Nursing, Physiotherapy and Medicine, Faculty of Health Sciences, University of Almería, 04120 Almería, Spain; 3 Instituto de Ciencia Molecular, Universitat de València, Catedrático José Beltrán, 2, 46980 Paterna, Spain; 4 Present Address: Department of Inorganic Chemistry, University of Granada, Av. Fuentenueva S/N, 18071 Granada, Spain; 5 Present Address: School of Chemistry, University of Birmingham, Edgbaston, Birmingham, B15 2TT UK

**Keywords:** Chemistry, Materials science

## Abstract

Photocatalysis process is a promising technology for environmental remediation. In the continuous search of new heterogeneous photocatalysts, metal–organic frameworks (MOFs) have recently emerged as a new type of photoactive materials for water remediation. Particularly, titanium-based MOFs (Ti-MOFs) are considered one of the most appealing subclass of MOFs due to their promising optoelectronic and photocatalytic properties, high chemical stability, and unique structural features. However, considering the limited information of the reported studies, it is a hard task to determine if real-world water treatment is attainable using Ti-MOF photocatalysts. In this paper, via a screening with several Ti-MOFs, we originally selected and described the potential of a Ti-MOF in the photodegradation of a mixture of relevant Emerging Organic Contaminants (EOCs) in real water. Initially, two challenging drugs (*i.e.*, the *β*-blocker atenolol (At) and the veterinary antibiotic sulfamethazine (SMT)) and four water stable and photoactive Ti-MOF structures have been rationally selected. From this initial screening, the mesoporous Ti-trimesate MIL-100(Ti) was chosen as the most promising photocatalyst, with higher At or SMT individual photodegradation (100% of At and SMT photodegradation in 2 and 4 h, respectively). Importantly, the safety of the formed by-products from the At and SMT photodegradation was confirmed. Finally, the At and SMT photodegradation capacity of MIL-100(Ti) was confirmed under realistic conditions, by using a mixture of contaminants in tap drinking water (100% of At and SMT photodegradation in 4 h), proven in addition its potential recyclability, which reinforces the potential of MIL-100(Ti) in water remediation.

## Introduction

There is no doubt that pharmaceuticals have efficiently increased life quality and expectancy. However, this exceptional step for humanity has nowadays led to an important environmental issue. Human and veterinary pharmaceuticals, and illicit drugs have been recently defined as Emerging Organic Contaminants (EOCs), being present in biota, sediments, effluents, and surface and ground water^1^. Despite their relatively low concentrations in natural waters (few ng·L^−1^–µg·L^−1^), EOCs are of particular concern for several reasons: (1) they have been detected in wastewater treatment plants (WWTPs) and hospitals effluents since these installations are not adapted to eliminate them; (2) many pharmaceuticals have been described recently as endocrine disruptors, raising many questions about risk to the environment and human health^2^; (3) they can be persistent, and their degradation products can be even more toxic than the parent products^3^, and (4) drug prescription is expected to sharply increase over the coming decades given the human population growth, the aging population and the drug medical coverage expansion. An increasing number of EOCs in the environment call to not only their monitoring and detection, but also, their efficient elimination.

Among the proposed technologies (*e.g.*, ion-exchange, adsorption and flotation, reverse osmosis)^4^, advanced oxidation processes (AOPs; *e.g.,* photocatalysis, Fenton reactions, sulphate radical mediated oxidations) have attracted a great deal of attention for the water treatment due to their simplicity and reproducibility^4,5^. In particular, heterogeneous photocatalysts such as TiO_2_, ZnO, Fe_2_O_3_, CdS, GaP, and ZnS have been proposed for the efficient degradation of a wide range of EOCs. However, some of them show typical drawbacks under the operating conditions, like toxicity, low photocurrent quantum yield due to electron hole recombination and/or low solar energy utilization efficiency, resulting from their narrow band gap.

In the continuous search of new semiconductor-based photocatalysts, metal–organic frameworks (MOFs) have recently emerged as a new type of photoactive materials for water remediation. MOFs are an outstanding class of micro/mesoporous coordination polymers, comprising inorganic nodes (*e.g.,* single-atoms, polynuclear clusters, infinite 1D chains) and organic polydentate linkers (*e.g.,* carboxylates, phosphonates, nitrogenated ligands) that assemble into multidimensional periodic lattices^6^. Because of their properties (biocompatibility, porosity, photoactivity, stability, etc.), MOFs have been studied for the degradation of pharmaceuticals. From the first report in the catalytic degradation of an industrial product (phenol) using a MOF in water in 2007^7^, considerable work has been published during the last few years. Particularly, titanium-based MOFs (Ti-MOFs) are considered one of the most appealing subclass of MOFs due to their promising optoelectronic and photocatalytic properties, high chemical stability, and unique structural features^8,9^. However, compared to other MOFs, the number of Ti-MOF structures is very limited^10^. Regarding the EOC removal, only the benchmarked Ti(IV)-aminoterephthalate MIL-125-NH_2_ has been reported for this application. In this regard, there are nearly 7 composites based on MIL-125-NH_2_ that have shown interesting performances in the EOCs photodegradation. However, prior to the potential use of Ti-MOFs in photoinduced water treatment, some issues need to be tackled, as the majority of the mentioned reports: (1) do not consider the MOF stability (leaching of its components) under working conditions; (2) some of the reported composites are based on highly toxic cations (*i.e.,* MIL-125-NH_2_@CdS)^11^; (3) photocatalytic tests are performed in distilled or Milli-Q water (far from the more complex real water samples); (4) need an equilibrium time (normally 1 h) to reach the EOC adsorption before irradiation or the addition of extra-species to be active (*e.g.,* Na_2_SO_4_); and/or *v)* study the degradation of just one contaminant, and not a mixture of them, as they are often found in real contaminated water. Although we recently reported a continuous flow system for the photodegradation of sulfamethazine (SMT) using MIL-125-NH_2_@Ag nanoclusters^12^, the photodegradation of a mixture of contaminants using MOFs and/or their recycling have never been investigated. Therefore, considering the limited information of the reported studies, it is a hard task to determine if real-world water treatment is attainable using Ti-MOFs as photocatalysts.

**Figure 1:**
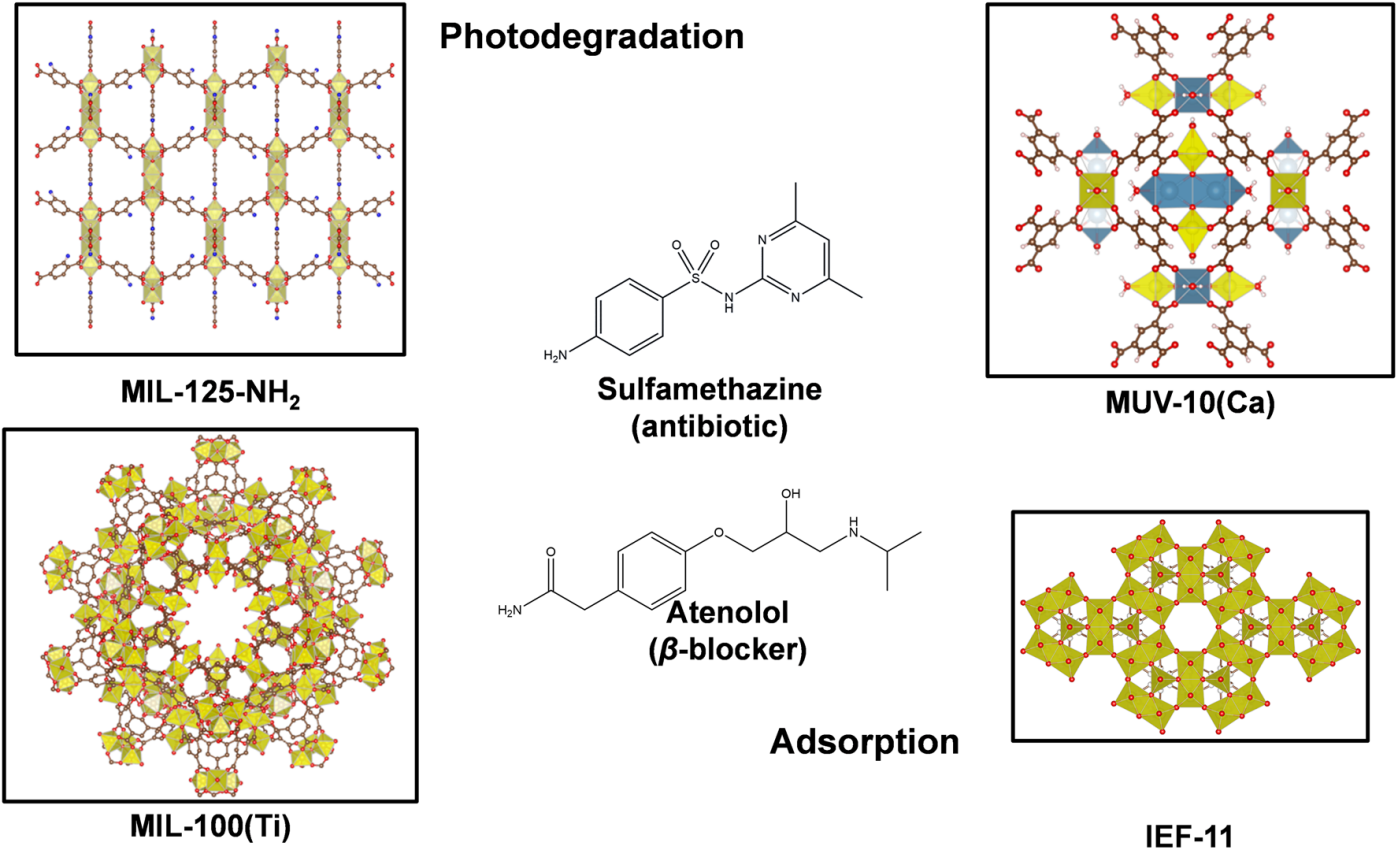
Schematic view of the structure of MIL-125-NH_2_, MUV-10(Ca), MIL-100(Ti) and IEF-11 (titanium polyhedra, calcium, oxygen, nitrogen, and carbon are represented in yellow, aegean blue, red, blue, and brown, respectively; hydrogen atoms are omitted for clarity). Structures of the antibiotic SMT and the *β*-blocker At are also given.

Accordingly, in the present investigation, we originally described the use of a Ti-MOF in the photodegradation of a mixture of relevant EOCs in real water. Initially, from the large panel of EOCs, and as a result of a thorough research (See Section “Selection of EOCs”), two challenging drugs (*i.e.*, the *β*-blocker atenolol (At) and the veterinary antibiotic sulfamethazine (SMT)) were chosen based on statistic data available in the literature (*i.e.,* exponentially increasing prescription, presence in water, toxic effects, potential development of bacterial resistances, and persistence; see below). Then, for the efficient degradation of these contaminants, we have rationally selected 4 previously reported photoactive highly stable Ti-MOF structures (Fig. 1). In a first step, a fast screening was carried out in tap water to select the most robust MOF with higher At or SMT individual photodegradation, identifying the mesoporous Ti-trimesate MIL-100(Ti) as the most promising photocatalyst. Subsequently, we have identified the formed by-products from the At and SMT photodegradation, considering their potential toxicity, in order to assess the impact of the MIL-100(Ti) treatment on the resulting water quality. Finally, to deeply evaluate the potential application of MIL-100(Ti) in water remediation, a photodegradation study was carried out under realistic conditions (*i.e.,* mixture of contaminants in tap drinking water, in absence of other co-catalyst/additive, without an adsorption equilibrium time) and considering the potential recyclability, which highlights the originality and interest of this work.

## Results and discussion

### Selection of EOCs

Among the commonly used 100 pharmaceuticals^13^, anti-hypertensive and antibiotic drugs are two of the most consumed groups. Arterial hypertension is one of the main chronic health problems, being extremely prevalent (20%) among the older adult population^14^. *β*-blockers are among the most consumed drugs used in arterial hypertension treatment^15^. During 2020, the consumption of *β*-blockers ranged around 24,495 (defined daily doses *per* 1,000 inhabitants *per* day)^16^. In particular At, a *β*-blocker drug primarily used to treat patients suffering from various heart disorders such as high blood pressure, chest pain (angina), migraines, and irregular heartbeats^17^, was ranked the 31st most prescribed drug in United States of America in 2014^18^. In addition, At cannot be fully metabolized by the human body, undergoing incomplete absorption (*ca.* 90%) and being largely excreted unchanged in the urine^19–21^, exponentially increasing its presence in water (from 0.35 to 2.21 mg·L^-1^)^22,23^. Further, the presence of At in water may cause severe toxic effects (*e.g*., dizziness, feeling tired, depression, chances of heart failure, shortness of breath, and cause bronchospasm), being also accumulated in breast milk, which is related to the immaturity of the renal function of neonate breast-fed infants^17,24^.

On the other hand, because of their importance for human and veterinary medicine, but also because of their persistence, sulfonamides, quinolones and trimethoprim are the most widely detected antibiotics in water^25^. Only in Spain, in 2020 the daily dose of prescribed antibiotics by the public health system were 19 *per* 1000 inhabitants *per* day according to the Spanish Agency for Medicines and Health Products. Among the most widely prescribed groups of antibiotics, we can find *β*-lactams (53.4%), macrolides and lincosamide (11.3%), quinolones (9.8%), tetracycline (8.0%), and sulfonamides and trimethoprim (2.5%)^26^. Regarding the veterinary use of antibiotics, in the European Union, the bestseller antibiotics were tetracyclines (30.4%), *β*-lactams (26.9%) and sulfonamides (9.2%)^27^. Particularly, the sulfonamide sulfamethazine (SMT), frequently used in pigs and cattle, has been detected in natural environments such as soil or water, ranging from a few ng to tens of mg *per* liter or kg of soil^28,29^. Large proportions of this compound is excreted unchanged in feces and urine given its incomplete metabolism^30^. Furthermore, the presence of SMT in water is associated with significant risks for humans due to the development of microbial resistances, even at low doses^31^.

### Screening of the SMT and At photodegradation and MOF stability

For the efficient removal of EOCs from water, we have selected 4 Ti-MOFs based on: (1) Ti-oxoclusters with redox and photocatalytic activity that could be exploited in the photodegradation of EOCs^10^, (2) their a priori remarkable hydrolytic stability (mostly tested in pure MilliQ water), (3) their exceptional porosity (see SI, Table S1**)**, in some cases compatible with the SMT and At dimensions (11 × 5 × 5 Å^3^, and 15 × 7 × 5 Å^3^, respectively; estimated by Vesta considering van der Waals radii), and (4) in terms of cost, they could be considered affordable for large scale production in the future^32^. The series comprises: (1) the **MIL-100(Ti)** or [Ti_3_(µ_3_-O)O(OH)_2_(BTC)_2_] (MIL: Materials Institute Lavoisier) based on the trimesate ligand (BTC), which combines a high chemical stability and mesoporosity (~ 25 & 29 Å, accessible via ~ 5 & 8.6 Å windows; S_BET_ ~ 1300 m^2^·g^−1^) with photoactive Ti_3_(µ_3_-O) metal-oxoclusters^33^; (2) the **MIL-125-NH**_**2**_ or [Ti_8_O_8_(OH)_4_(BDC-NH_2_)_6_] built from Ti-oxoclusters coordinated to the 2-aminoterephthalate ligand (BDC-NH_2_)^34^, exhibiting a high microporosity (~ 13 & 6 Å, accessible via ~ 6 Å windows; S_BET_ ~ 1400 m^2^·g^−1^) and robustness; (3) the **MUV-10(Ca)** or [Ti_3_Ca_3_(µ_3_-O)_3_(BTC)_4_(H_2_O)_6_] porous solid (MUV: Materials of the University of Valencia) built from the interlinking of fully deprotonated trimesate anions and tetranuclear Ti^IV^_2_Ca^II^_2_(µ_3_-O)_2_(H_2_O)_4_(CO_2_)_8_ clusters^35^, with an important porosity (~ 10 Å, accessible via ~ 5 Å windows; S_BET_ ~ 1000 m^2^·g^−1^); and (4) the recently reported **IEF-11** or [Ti_2_O_3_(SQ)] (IEF: IMDEA Energy Framework)^36^, based on photo/redox 2D titanium layers and the squarate (SQ, 3,4-dihydroxycyclobut-3-ene-1,2-dionate) as organic linker (see detailed properties in Table S1), with small porosity (~ 4.5 Å; S_BET_ ~ 120 m^2^·g^−1^) but an exceptional hydrolytic stability**.**

**Figure 2:**
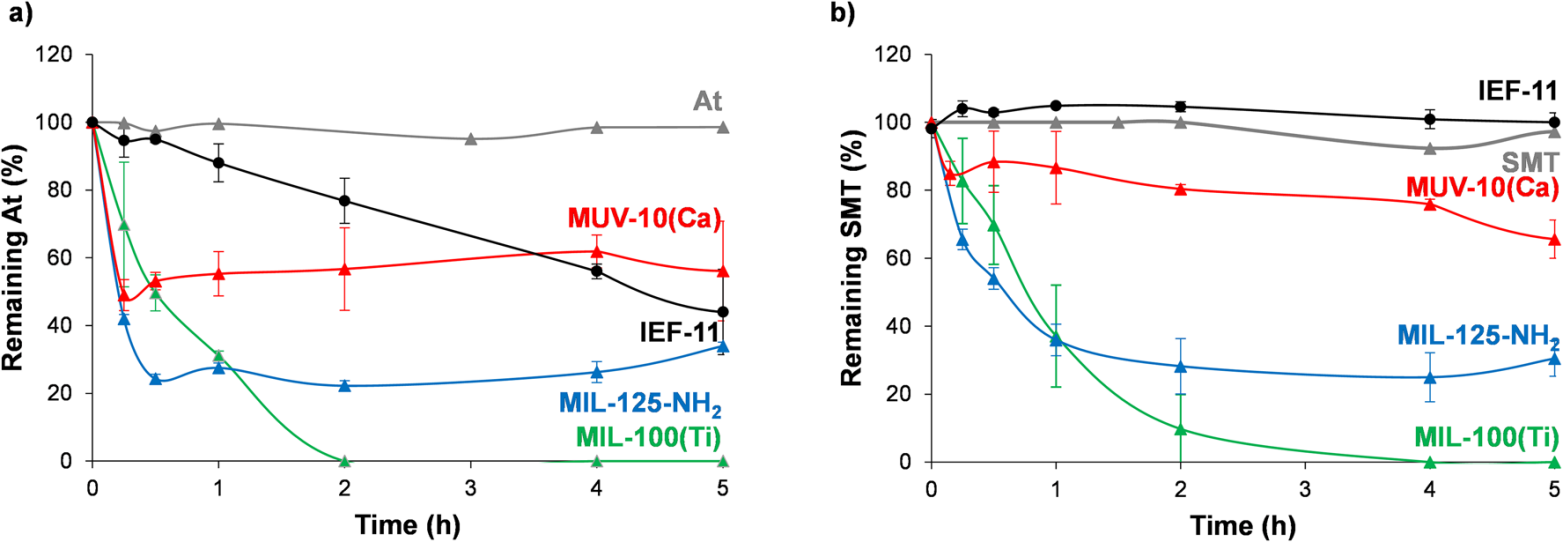
Comparative photodegradation evolution of At (**a**) and SMT (**b**) using different Ti-MOFs. For clarity, degradation of MOFs is omitted here (although included in the **SI, Figure S2&S3**).

First, the SMT and At photodegradation capacity and the matrix chemical stability of the selected Ti-based MOFs were studied by using similar At and SMT concentrations normally reported in the environment (5 ppm^37^, and 35 ppm^38,39^, respectively). The At and SMT photodegradation capacity strongly depends on the used MOF, decreasing in the following order: (At) MIL-100(Ti) > MIL-125-NH_2_ >  > IEF-11 > MUV-10(Ca); and (SMT) MIL-100(Ti) > MIL-125-NH_2_ >  > MUV-10(Ca) > IEF-11 (Fig. 2).

Interestingly, these results highlight the remarkable ability of the MIL-100(Ti) to remove both EOCs, eliminating 100% of At and SMT in only 2 and 4 h, respectively. The plateau of degradation is reached only after 1 h in all studied materials, except for the continuous At degradation of IEF-11, and SMT and At degradation of MIL-100(Ti) where it took only 2 h. Factors like MOF structure/porosity (pore accessibility, surface, volume, tortuosity, connectivity, particle size, etc.) and nature (ligand, cluster structure, band gap, external surface, etc.) might influence the pharmaceuticals degradation capacity. For instance, compared with the rest of studied Ti-MOFs, the lower *accessibility* of IEF-11, with a pore and window size of 4.5 Å, might hamper the accessibility of At and SMT and, therefore, their photodegradation. In fact, there is no SMT degradation when using IEF-11 and the kinetics of degradation of At is *ca.* 11-fold lower than MIL-100(Ti) (Table 1). Regarding the chemical nature of MOFs, there is not a direct relationship between the calculated *band gap* values (Fig. S1) and the photocatalytic capacity of these materials in the studied reaction.

**Table 1:** Total At and SMT photodegradation (after 5 h, %), MOF degradation (%), and kinetic constant (M^-1^·h^-1^) for all studied materials.

MOF	Photodegraded At (%) MOF (%)	Kinetic constant (M^−1^·h^−1^) R^2^	Photodegraded SMT (%) MOF (%)	Kinetic constant (M^−1^·h^−1^)
MIL-100(Ti)	100 ± 0 3.8 ± 0.1	12,199 0.985	100 ± 0 3.2 ± 0.3	112,013 0.964
MIL-100(Fe)	65.0 ± 4.4 9.7 ± 1.5	247 0.957	66.4 ± 10.4 1.0 ± 0.1	24,937 0.996
MIL-125-NH_2_	66.0 ± 1.1 38.8 ± 1.2	122,555 0.985	69.5 ± 5.2 39.3 ± 0.4	70,213 0.958
MUV-10(Ca)	43.9 ± 14.7 41.0 ± 1.9	64,034 0.995	34.4 ± 5.6 36.1 ± 6.4	4946 0.896
IEF-11	56.0 ± 12.6 0.78 ± 0.01^a^	1124 0.982	0.00 ± 0.01 0.35 ± 0.01^a^	–

^a^Value corresponding to 24 h.

For comparison, the At and SMT adsorption capacity of the studied Ti-MOFs was performed in absence of light. As in the photocatalytic studies, the adsorption of At and SMT strongly depends on the MOF, decreasing in the following order: (At) MIL-125-NH_2_ > MIL-100(Ti) > MUV-10(Ca); and (SMT) MIL-125-NH_2_ > MIL-100(Ti) ~ MUV-10(Ca) (Figs. S2–S4). Unlike its remarkable pharmaceuticals photodegradation capacity, MIL-100(Ti) can eliminate by adsorption only 13.5 ± 3.9% of At and 0 ± 3.9% of SMT after 5 h. Again, in a view of providing a comparison, the same experiment was performed using the Fe-based MIL-100(Fe) analogue, obtaining similar adsorption capacities for At (8.2 ± 5.5%) and SMT (14.2 ± 8.0%) than the MIL-100(Ti). The formation of interactions between the At and the MIL-100(Ti) is evidenced by Fourier transform infrared spectroscopy (FTIR) by comparing the spectra of free At to that of the empty and the At loaded material (At@MIL-100(Ti)). Specifically, there is a shift in the wavelengths of pure At (from 2964 and 2922 cm^−1^ to 2955 and 2925 cm^−1^ for At and At@MIL-100(Ti), respectively; see SI, Figure S5**),** characteristic of the *ν*(C–H) group of the C9 (near the ether group C9–O–benzene)^40^. In contrast, the MIL-125-NH_2_ was able to adsorb up to 66.6 ± 1.1% of At and 50.2 ± 3.4% of SMT after 5 h. However, the release of the previously adsorbed EOCs was evidenced after 1 h, probably related with the degradation of the framework during the drug adsorption process (SI, Figure S2&S3). This is supported by the significant leaching of the linker (*ca.* 18% in both SMT and At adsorption after 5 h), confirmed by the similar FTIR spectra of the drug-loaded MIL-125-NH_2_ and the free linker H_2_BDC-NH_2_ (SI, Fig S6), and the amorphization of the MIL-125-NH_2_ framework after the adsorption process (SI, Figs. S7&S8).

On the other hand, it is imperative to get fast degradation kinetics in order to achieve more efficient removal processes. The comparison of the At and SMT degradation kinetics has been performed through the fitting of the data to a second order kinetics according to Eq. (1) (see experimental section and SI, Figure S9). Although the data have also been fitted to a zero and first order kinetics, a better correlation was found using the second order. This is in agreement with previously reported photoactive MOFs and composites (*i.e.,* MIL-125-NH_2_ and AgNC@MIL-125-NH_2_ in the degradation of SMT or methylene blue)^12^. The best degradation rate is obtained when using MIL-125-NH_2_ in the At degradation, with a *k* value of 122,555 M^−1^·h^−1^, which is *ca.* 2, 10 and 109-fold higher than MUV-10(Ca), MIL-100(Ti), and IEF-11, respectively. In the case of SMT, the best degradation rate is obtained for MIL-100(Ti), with a *k* value of 112,013 M^−1^·h^−1^, which is *ca.* 2, 5, and 23-fold higher than MIL-125-NH_2_, MIL-100 (Fe), and MUV-10(Ca), respectively. Finally, when evaluating the kinetics of the photocatalytic process using MIL-100(Ti) and MIL-125-NH_2_, the *particle size* and the *external surface* may also play important roles. It is generally accepted that smaller particles might favor the catalytic reactions. Tentatively, we can argue that the smaller MIL-125-NH_2_ particles (*ca.* 240 nm with an estimated external surface of 225 mg^2^·g^−1^ by t-plot method (p/p_0_ from 0.3 to 0.6)) than MIL-100(Ti) (> 1 µm with an external surface of 195 mg^2^·g^−1^) will favor the transport of EOCs and degradation products.

Aside from the EOCs elimination capacity, the chemical stability of the framework is a limiting parameter in water decontamination processes. The point is to avoid an extra contamination of water due to the leaching of the MOFs constituents (*e.g*., ligands, metals). Therefore, and in contrast with the vast majority of reports on this topic, we have here considered the possible release of the MOF’s building blocks. The chemical stability of a coordination network mainly depends on the strength of the metal to linker bonds, which can be estimated according to the hard and soft acids and bases (HSAB) principle^41^, and the competition with reactive species found in the solution. Thus, the release of the constitutive ligand was monitored by HPLC and the crystalline structure was checked by XRPD. At this point, it should be noted that the degradation of the frameworks was not evaluated via the quantification of the released metallic species in solution as, under the working conditions (contaminated tap water, pH = 8.2) and according to the Pourbaix diagram^42^, the vast majority of the degraded metallic species would be precipitated (*i.e.,* TiO_2_), underestimating the MOF degradation. Through the linker release, MOFs degradation could be also overestimated, as it can be adsorbed in the porosity of the framework (*e.g*., poorly activated/purified solids). However, we should rule out the release of any species that could be toxic, which in imperative for water decontamination. During the At and SMT photodegradation, the chemical stability of the framework in At or SMT-contaminated tap water decreased in the following order: (At) IEF-11 > MIL-100(Ti) >  > MIL-125-NH_2_ ~ MUV-10(Ca); and (SMT) IEF-11 > MIL-100 (Ti) >  > MUV-10(Ca) ~ MIL-125-NH_2_ (Table 1). Note here that the stability ranking is very similar for At and SMT, ruling out an important effect of the drug nature on the MOF degradation. In addition, the XRPD patterns of the At- and SMT-containing MOFs evidenced that the At or SMT loading process does not alter the crystalline structure of MIL-100(Ti) and IEF-11, but there is an important peak broadening in the MUV-10(Ca) and MIL-125-NH_2_ materials, consistent with a crystallinity loss (SI, Figure S7&S8). Remarkably, MIL-100(Ti) and IEF-11 show a high chemical stability under the working conditions with only *ca.* 3 and 0.7% degradation, respectively, after 5 h in contact with the contaminants’ solutions.

Considering the above results, the most promising photocatalyst for the At and SMT removal in water is MIL-100(Ti), demonstrating not only an exceptionally high and fast EOCs degradation (100% in 2 and 4 h, respectively), but also a moderate matrix degradation (*ca.* 9%). This is particularly important considering that the median oral lethal dose (LD_50_) for H_3_BTC is *ca.* 4 times lower than the one for SMT and At (in rats, LD_50_ is 8.4, 2 and > 2 g·kg^−1^ for H_3_BTC, SMT and At). Therefore, the proposed photodegradation method is efficiently improving the water quality within a short time. The results on the photodegradation of At and SMT using MIL-100(Ti) are on the range or even overpass the results obtained with other MOFs, MOF composites or other materials (SI, Table S2).

At this point, in an attempt to rationalize the complex process involved in the At and SMT photodegradation using MIL-100(Ti), we have explored the photocatalytic performance of the isostructural iron analogue MIL-100(Fe) under equal conditions. It should be pointed that even the different band gap values of the Ti and Fe based MIL-100 materials (3.48 and 2.73 eV, respectively, SI, Figure S1), both suspensions were irradiated in all the UV–vis range. As expected, the Ti-based MIL-100 is more efficient than its Fe counterpart, with a 100% and *ca.* 65% of At and SMT degradation after 5 h-irradiation, evidencing the crucial role of the titanium trimeric clusters. When comparing the kinetic of the process (Table 1), the degradation rates of At and SMT by MIL-100(Ti) is *ca.* 49 and 4.5-fold higher than the Fe isostructural MOF. Both materials are chemically stable under the studied conditions, with only *ca.* 10 and 1% of MIL-100(Fe) degradation in At and SMT solutions, respectively. However, in the case of MIL-100(Fe), the degradation of the framework seems to be affected by the nature of the contaminant, being favored in the presence of At. The amide of At may preferentially interact with the Fe metal sites than with the Ti ones (more oxophilic). Finally, the accessible porosity of the framework is not a determining factor when comparing the Fe and Ti-based MIL-100 materials. Although the accessible porosity of MIL-100(Fe) is double than the one of MIL-100(Ti) (SI, Table S1), this last material is the most effective in At and SMT degradation.

### Photodegradation products using MIL-100(Ti)

The hazard of these EOCs does not only relay on their concentration or toxicity, but also their metabolites or degradation products, which can sometimes be more harmful than the parent compounds^43^. The degradation products of At and SMT using MIL-100(Ti) were thus independently analyzed. Previous studies have described the photochemical behavior of At and SMT, determining that the degradation occurs through the cleavage at various positions (Figure S10)^44^. The identification of the photodegradation products of At and SMT formed after 5 h-irradiation of the At@MIL-100(Ti) and SMT@MIL-100(Ti) systems in aqueous medium was carried out using UHPLC/MS, supported with fragmentation patterns obtained from MS/MS experiments. Although the determination of the EOCs degradation pathway is out of the scope of this work, we have successfully identified some intermediates deduced from their estimated molecular weight, allowing to assess the potential toxicity of the resulting products.

Particularly for At, cleavage of the side chain and the addition of the hydroxyl group to the parent compound were found to be the two main degradation pathways (Figures S10&S11). Thus, the fragment ion *m/z* 134 (3-(isopropyl)propane-1,2-diol) was attributed to the product from the chain cleavage, and the ions *m/z* 167 2-(2,4-dihydroxyphenyl)acetamide and 2-(3,4-dihydroxyphenyl)acetamide to the chain cleavage and oxidation. These photodegradation pathways and intermediates have been frequently reported in the photodegradation of At^45–47^.

On the other hand, in the SMT photodegradation study, the fragment ion *m/z* 216.7 (*N*-(4,6-dimethylpyrimidin-2-yl)benzene-1,4-diamine) was attributed to the product from SO_2_ extrusion, a phenomenon frequently shown in sulfonamides (Figs. S10&S12)^48^. The attack of a hydroxyl radical at the C–N bond of the benzene ring might result in the fragment derived from the pyrimidinyl portion (m/z 124), leading to the formation of the 2-amino-4,6-dimethoxypyrimidine product. The cracking of the N-containing benzene ring is attributed to the formation of the fragment ions *m/z* 197 (4-amino-*N*-(iminomethylene)benzenesulfonamide) and m/z 213 (4-(2-imino-4,6-dimethylpyrimidin-1(2*H*)-yl)cyclohexamine), as previously reported^49^.

Importantly, regarding the potential toxicity of the degradation products, neither of the intermediated formed through At and SMT photodegradation exhibit acute toxicity^45^, supporting then the significant improvement of the resulting water quality.

### Combined EOCs photodegradation using MIL-100(Ti)

**Figure 3:**
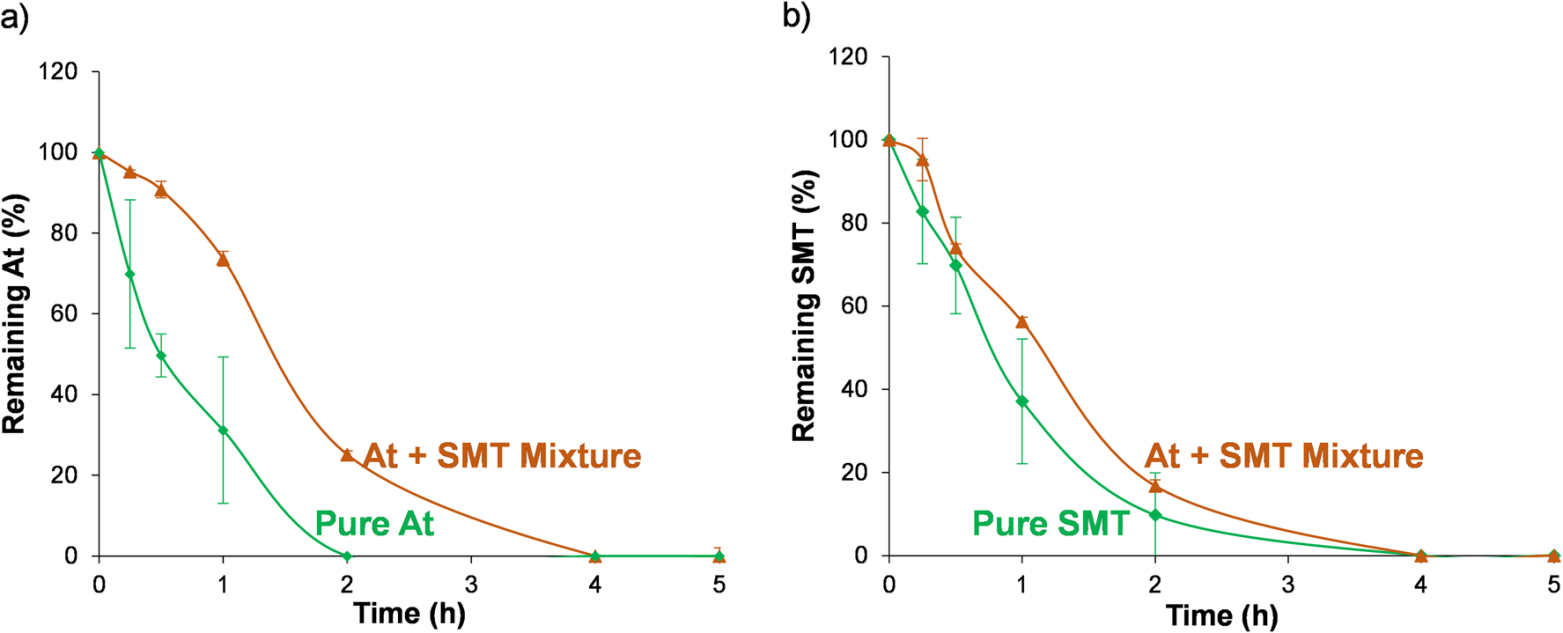
Effect of the mixture (brown, triangles) or single contaminants (green, diamonds) on the photodegradation of At (**a**) and SMT (**b**) using MIL-100(Ti).

In real water environments, physicochemical conditions are not static (e.g., presence of more than one pollutant with a different range of concentrations, or the pH of natural waters are normally found between 5 and 8)^50,51^. Therefore, the target of an efficient photocatalyst should be the elimination of most of the EOCs present in this water in a range of different conditions (contaminants and catalyst concentration and contaminated water pH). In this context, first the At and SMT removal by MIL-100(Ti) was simultaneously investigated using a mixture of both contaminants in tap water (initial pH = 8.2). In this sense and to the best of our knowledge, there are no reported examples of the combined photodegradation of water EOCs using MOFs, but only the evaluation of their combined adsorption^52^. Remarkably, when both pollutants are present in solution, the time required to fully degrade the SMT is similar to the single SMT removal (100% of the SMT is removed in only 4 h, Fig. 3). In contrast, the At elimination is slowed down, and the time required to remove 100% of the At is doubled (from 2 to 4 h). The kinetics of the process is modified in both reactions, being the degradation rate reduced up to 4.5 and 2.4-fold for At and SMT, respectively (Table 2). This could be explained by the competitive degradation of SMT and At, where the SMT is favored over the At photodegradation. Regarding the MOF stability, in presence of both EOCs, the leaching of the H_3_BTC linker is maintained, indicating the potential of the MIL-100(Ti) in the photodegradation of EOCs in water. In a further step to real conditions, other factors such as initial concentration of At and SMT, catalyst amount, and pH, were analyzed. The effect of the *initial concentration of contaminants* was studied using different At and SMT initial concentrations (from C_0_ = 35 and 5 ppm to 17.5 and 2.5 ppm, respectively). MIL-100(Ti) degraded most of the SMT (95%) at low initial concentration, while the degradation efficiency of At decreased from 100% (C_0_ = 35 ppm) to 15% (C_0_ = 17.5 ppm) after 5 h irradiation. This phenomenon suggests that MIL-100(Ti) efficiently increases with EOCs concentration, which has great significance in more dangerous (toxic) environments. In agreement, no changes in the crystallinity or MIL-100(Ti) particles morphology are observed (SI, Figure S12).

**Table 2:** Total combined At and SMT photodegradation (after 5 h, %), MOF degradation (%), and kinetic constant (M^−1^·h^−1^) for MIL-100(Ti).

EOCs	Photodegraded At (%)	Kinetic constant (M^−1^·h^−1^) R^2^	Photodegraded SMT (%)	Kinetic constant (M^−1^·h^−1^)	MIL-100(Ti) degradation (%)
At	100 ± 0	12,199 0.985	–	–	3.8 ± 0.1
SMT	–	–	100 ± 0	112,013 0.964	3.3 ± 0.3
At + SMT	100 ± 0	2755 0.946	100 ± 0	45,835 0.966	1.34 ± 0.29

On the other hand, when the *amount of catalyst* is reduced by half (from 4 to 2 mg), a high SMT removal (*ca.* 83%) is achieved, while the At removal capacity is considerably reduced (only *ca.* 44% in 5 h). In terms of MOF stability, the XRPD patterns showed that changes in contaminants concentration do not affect the crystallinity of MIL-100(Ti), while an important amorphization is observed when less amount of MIL-100(Ti) is used (SI, Figure S12). These results are supported by FESEM images, with important morphological changes in MIL-100(Ti) when the amount of MOFs is halved.

Finally, the *effect of pH* in At and SMT degradation was studied, by changing from an initial pH of contaminated water from 8.2 to 6.4 and 5.5. Under acidic conditions (pH = 6.5 and 5.5), the SMT elimination levels are maintained (*ca.* 90 and 91% of SMT degradation, respectively), while the At degradation capacity is strongly affected (only 27 and 9% of At is degraded in 5 h, respectively). Considering the pk_a_ of SMT (pK_a1_ = 2.65-aromatic amine; pK_a2_ = 7.65-sulfonamide) and the pka of At (9.6-amine), SMT is predominantly neutral (pH = 6.4 and 5.5) or protonated (pH = 8.2), while At is always protonated (pH = 5.5–8.2). Therefore, the protonation or deprotonation of the EOCs will not strongly affect the intermolecular electrostatic attraction between EOCs and MIL-100(Ti). However, considering that one of the interactions of the At with the MOF is through the central O of the At (according to FTIR, see above), it could be hypothesized that, as the attraction between the drug and the framework is stronger when the initial pH is higher, leading into a higher photocatalytic performance. The XRPD patterns demonstrated an amorphization under acidic conditions (SI, Figure S13). From the practical point of view, and although an optimization of the process is needed, we can conclude that the optimal working conditions for MIL-100(Ti) in SMT and At degradation are high EOCs concentrations (35 and 5 ppm, respectively), 4 mg of catalyst and alkaline waters.

### Cyclability of MIL-100(Ti)

**Figure 4:**
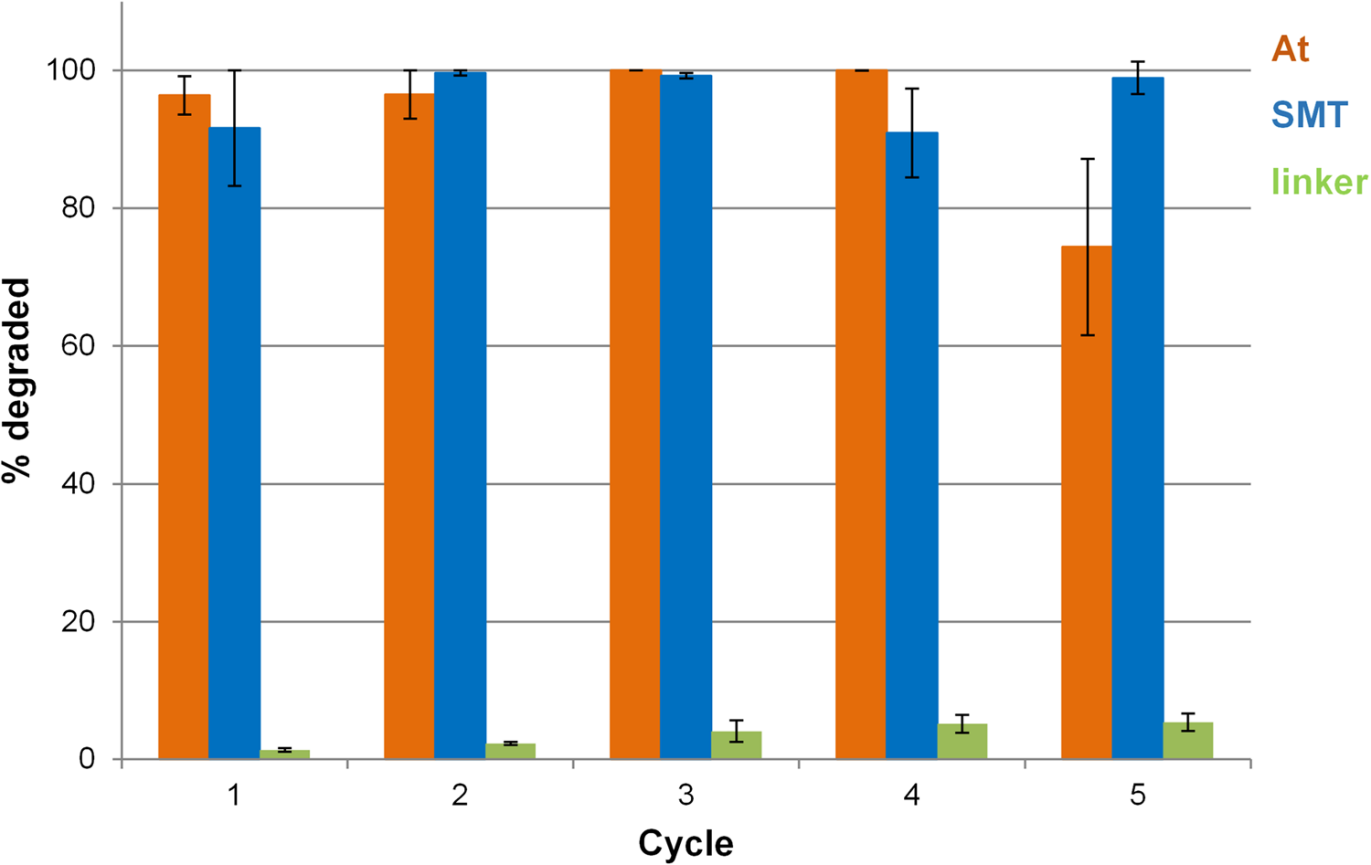
Cyclability tests of mixtures of SMT and At photodegradation in tap water using MIL-100(Ti).

The efficiency of a catalyst is significantly enhanced if the material can be re-used for several photodegradation cycles. After 5 h-irradiation, MIL-100(Ti) was recovered from the treated water by centrifugation, and resuspended in fresh contaminated water (mixture of contaminants). Upon 5 photodegradation cycles, MIL-100(Ti) efficiently removed SMT and At without a significant decrease on the efficiency (Fig. 4). Note here however that, upon the first and second cycles, an important peak broadening, consistent with a partial amorphization, is observed in the XRPD patterns (SI, Figure S14), leading to a completely amorphous solid after the third cycle. In contrast, the chemical integrity of MIL-100(Ti) is kept after the 5 cycles, with only 5% of the linker released. One could suggest that upon photocatalytic cycles, the chemical composition of the MIL-100(Ti) is almost kept, while the creation of defects leads to the loss of the long-range order, while improving the quality of water.

## Conclusions

The screening of 4 robust Ti-MOFs for the removal of EOCs in tap water has allowed the selection of an extremely efficient photocatalyst, the mesoporous titanium trimesate MIL-100(Ti). Here, we present the successful photodegradation of the challenging EOCs, SMT and At, by this material, able to efficiently eliminate SMT and At (100% removal at 2 and 4 h, respectively) from tap water without an important chemical degradation. In addition, the generation of lower potential toxic products upon photodegradation supports the significant improvement of the quality of water. Further, when working with a contaminants mixture, the ability of MIL-100(Ti) to remove SMT is kept intact, while the At photodegradation is slowed-down, as a consequence of the drug competition. Upon 5 photodegradation cycles, MIL-100(Ti) efficiently removes both SMT and At, envisioning the future real application of this MOF on the removal of SMT and At as well as other widespread EOCs in environmental remediation.

## Methods

All reactants were commercially obtained and used without further purification. The synthesis of starting materials was performed following similar synthetic procedures as previously reported (see below).

### Synthesis of MOFs

#### Synthesis of MIL-100(Ti) or [Ti_3_(µ_3_-O)O(OH)_2_(BTC)_2_]^33^

Ti_6_ (7.2 mg, 24 mmol of Ti) and trimesic acid (H_3_BTC, 25.0 mg, 120 mmol) were dispersed in 3 mL of a mixture of acetonitrile:tetrahydrofuran (ACN:THF, 3:1, v/v%) in a glass vial. Subsequently, 250 mL of acetic acid (180 equiv.) were added, and the mixture was sonicated to get a homogeneous suspension. The vial was placed in the oven at 160 ºC for 48 h. After cooling down to room temperature (RT), the white microcrystalline powder was recovered by centrifugation at 5000 rpm for 2 min, rinsed with fresh *N*,*N*-dimethylformamide (DMF) and methanol (MeOH) and further purified by Soxhlet extraction with hot ethanol (EtOH) or MeOH for 8 h. The solid was then allowed to dry under vacuum at RT.

#### Synthesis of MIL-100 or [Fe_3_O(H_2_O)_2_OH(BTC)_2_]^53^

FeCl_3_·6H_2_O (2.7 g, 10 mmol) and ethyl ester 1,3,5-benzenetricarboxylic (2.24 g, 6.66 mmol) were dispersed in 50 mL of deionized H_2_O. The mixture was placed in a Teflon-lined autoclave at 130 °C for 3 days. Then, the orange solid was recovered by filtration and washed with absolute EtOH (3 × 10 mL). The solid was then suspended in 1 L of EtOH, refluxed under stirring for 3 h and then, the same procedure was performed in deionized water. Further activation was carried out by redispersing the solid in 100 mL of KF aqueous solution (0.1 M). The mixture was kept under magnetic stirring for 2 h and 40 min under ambient conditions. Immediately after, the solid was washed with water at room temperature by suspending the solid in 250 mL of water for 4 h. The solid was recovered by filtration.

#### Synthesis MUV-10(Ca) or [Ti_3_Ca_3_(µ_3_-O)_3_(BTC)_4_]^35^

H_3_BTC (125 mg, 595 µmol) and CaCl_2_·6H_2_O (26.8 mg, 120 µmol) were dissolved in a mixture of 12 mL of dry DMF and 3.5 mL of acetic acid in a 25 mL Schott bottle. Subsequently, titanium(IV) isopropoxide (36 µL, 120 µmol) was added to the clear solution. The bottle was sealed and heated in an oven at 120 ºC for 48 h. After cooling down to RT, the microcrystalline powder was recovered by centrifugation at 5000 rpm for 2 min and rinsed with fresh DMF, water and MeOH several times and further purified by Soxhlet extraction with hot EtOH or MeOH for 8 h. The solid was then allowed to dry under vacuum at RT.

#### Synthesis of MIL-125-NH_2_ or [Ti_8_O_8_(OH)_4_(BDC-NH_2_)_6_]^34^

2-Aminoterephthalic acid (H_2_BDC-NH_2_, 1.38 g, 7.6 mmol) was dissolved in 20 mL of DMF and 5 mL of MeOH at RT under stirring. The mixture was placed in a round bottom flask (50 mL) equipped with a condenser and was warmed at 100 °C under air. When the mixture reached the temperature of 100 °C, 1.5 mL of titanium(IV) isopropoxide (1.5 g, 5.1 mmol) was added. The mixture was kept under magnetic stirring and heated for 72 h at 100 °C under air. The yellow solid obtained was filtered and washed with DMF at RT.

The as-synthesized dried product was dispersed at RT in DMF under stirring overnight (on, 100 mL of DMF *per* g of product). Then, the same procedure was repeated once using MeOH instead of DMF for 4 h.

#### Synthesis of IEF-11 or [Ti_2_O_3_(SQ)]^36^

Finely ground squaric acid (H_2_SQ, 255 mg, 2.24 mmol) was suspended in 8.2 mL of isopropanol in a 25 mL round-bottom flask, magnetically stirred for 5 min at RT and sonicated in an ultrasound bath for another 5 min. Then, glacial acetic acid (6.4 mL, 111.9 mmol) was added and sonicated again for 15 min. Then, titanium(IV) butoxide (762 μL, 2.24 mmol) was slowly added to the previous solution under stirring and then, heated at 50 °C for 15 min. The resulting orange suspension was transferred into a 23 mL Teflon-lined steel autoclave, sealed and heated at 120 °C for 48 h with heating and cooling ramp of 1.5 °C·min^−1^. The resulting orange-brown solid was filtered under vacuum, washed with isopropanol and dried in open air at RT. Finally, the sample was heated at 200 °C for 24 h in order to remove the remaining acetate moieties from the surface of the solid.

### Experimental techniques

Fourier transform infrared spectroscopy (FTIR) studies were recorded using a Nicolet 6700 FTIR thermo scientific spectrometer in the 400–4000 cm^−1^ region. X-ray powder diffraction (XRPD) patterns of all samples were collected in an Empyream PANALYTICAL diffractometer, equipped with a PIXcel3D detector and a copper radiation source (Cu K*α*, *λ* = 1.5406 Å), operating at 45 kV and 40 mA. Profiles were generally collected in the 3º < 2*θ* < 35º range with a typical step size of 0.013º and 40 s of acquisition.

N_2_ isotherms were obtained at 77 K using a TriStar II Plus Instruments. Before the measurement, samples were evacuated under vacuum at 150 ºC 16 h for MIL-100(Ti) and MUV-10(Ca), 200 °C 16 h for MIL-125-NH_2_, and 130 ºC 3 h for MIL-100(Fe). Specific surface area was determined by applying Brunauer, Emmett & Teller equation (BET) in the relative pressure interval p/p_0_ = 0.01–0.3 (being p_0_ the saturation pressure). External surface area was calculated by t-plot method in the relative pressure interval p/p_0_ = 0.3–0.6). FESEM (Field Emission Scanning Microscope) JEOL JSM-7900F was used to obtain high resolution images of nanomaterials with magnifications from 20 to 1,000,000x and secondary electrons detector.

*Different organic molecules were analyzed by HPLC:* the amount of degraded sulfamethazine (SMT) and atenolol (At), as well as the released corresponding linkers (H_2_BDC-NH_2_ or H_3_BTC), was determined using a reversed phase HPLC Jasco LC-4000 series system, equipped with a photodiode array (PDA) detector MD-4015 and a multisampler AS-4150 controlled by ChromNav software (Jasco Inc, Japan). A Purple ODS reverse-phase column (5 μm, 4.6 × 150 mm, Análisis Vínicos, Spain) was employed. For the quantification of all chemical species, isocratic conditions were used. The flow rate was 1 mL·min^-1^, and the column temperature was fixed at 298 K. In all cases, the injection volume was 30 μL. The mobile phase was based on a mixture of 50:50 MeOH:phosphate buffered solution (PBS, 0.04 M, pH = 2.5) for H_2_BDC-NH_2_ and H_3_BTC ligands analysis, with a retention time (rt) and an absorption maximum of 2.84 min and 228 nm, and 3.51 min and 225 nm, respectively. SMT was analyzed using a mixture of 35:65 ACN:water, with a rt of 2.7 min and an absorption maximum of 263 nm. At was analyzed using a mixture of 10:90 ACN:PBS (0.04 M: pH = 2.5), rt = 4.81 min and λ = 227 nm.

*Preparation of the PBS* (0.04 M, pH = 2.5): 0.02 mol (2.4 g) of NaH_2_PO_4_ and 0.02 mol (2.84 g) of Na_2_HPO_4_ were dissolved in 1 L of Milli-Q water. The pH was then adjusted to 2.5 with H_3_PO_4_.

### Adsorption and/or photocatalytic degradation of SMT and/or At from water

The adsorption/photocatalytic activity of Ti-MOFs was evaluated in terms of elimination of both EOCs, SMT and At. In a typical experiment, 4 mg of MOF (MIL-100(Ti or Fe), MUV-10(Ca), MIL-125-NH_2_ or IEF-11) were suspended in 4 mL of a SMT or At tap-aqueous solution (5 ppm^54^, and 35 ppm^22,23^, respectively; according with the concentration of SMT and At found in the environment). Adsorption/photodegradation reactions were performed under magnetic stirring. At certain intervals (0.25, 0.5, 1, 2, 4, and 5 h), an aliquot of 100 μL was collected by centrifugation for HPLC analysis (SMT, At and the corresponding ligand). All experiments were performed at least in triplicate to ensure statistically reliable results. The crystallinity of the remaining solids was analyzed by XRPD. First of all, the stability of an aqueous solution of SMT and At was studied under UV–vis light in absence of the photocatalyst. It was verified that SMT and At were not degraded (after 5 h) under UV–vis light irradiation.

*SMT and At photodegradation products:* Particularly in the At and SMT photodegradation studies, after 5 h of UV–vis irradiation, an aliquot was analyzed by mass quadrupole spectrometry coupled to ultra-high performance liquid chromatography (UHPLC) to determine the final products of the photodegradation. Standard samples of SMT and At were measured to deduce through comparison the mass parents obtained.

The photodegradation experiments were performed in a glass photoreactor equipped with a 300 W Xe lamp (Oriel Instrument OPSA500) under open air at RT, with the samples stirred and placed at a fixed distance of 21 cm from the irradiation source. It must be pointed that prior to irradiation it is not necessary to stir the suspension until the adsorption–desorption equilibrium is reached.

Although the selected Ti-based MOFs show a different experimental band gap (3.48, 2.73, 3.63, 2.53, and 2.40 eV for MIL-100(Ti), MIL-100(Fe), MUV-10(Ca), MIL-125-NH_2_, and IEF-11, respectively; values obtained by the Tauc Plot, Supporting information-SI Figure S1)^55^, we have irradiated all samples under the full UV–vis spectra. It should be noted that the use of a glass photoreactor may minimize the UV irradiation.

*Combined At and SMT adsorption and/or photodegradation capacity of MIL-100(Ti):* In an attempt to reproduce real water conditions, the same procedure was performed in a mixture of At and SMT in tap water. 4 mg of MIL-100(Ti) were suspended in 4 mL of a mixture of SMT (5 ppm) and At (35 ppm) tap-aqueous solution. Adsorption/photodegradation reactions were performed under stirring. At certain intervals (0.25, 0.5, 1, 2, 4, and 5 h), an aliquot of 100 μL was collected by centrifugation for HPLC analysis (SMT, At and H_3_BTC ligand). If necessary, pH was adjusted with diluted solutions NaOH and HCl. All experiments were performed at least in triplicate to ensure statistically reliable results. The crystallinity of all the remaining solids was analyzed by XRPD.

*Photodegradation kinetics:* To shed some light on the At and SMT degradation kinetics and to gain further understanding on the involved mechanism, the first 2 h of At and SMT degradation were fitted to a second order kinetics according to Eq. (1),

1 $$\frac{1}{\left[ A \right]} = \frac{1}{{\left[ A \right]_{0} }} + kt$$

where *[A]* and *[A]*_*0*_ is the remaining concentration of contaminant (M, mol·L^−1^) at the time *t* (h) and the initial contaminant concentration, respectively, and *k* is the second order kinetic constant (M^−1^·h^−1^).

*Recyclability of MIL-100(Ti)*: The recyclability of MIL-100(Ti) was studied repeating the same procedure above described. 4 mg of MIL-100(Ti) were suspended in 4 mL of a mixture of At (35 ppm) and SMT (5 ppm) tap-aqueous solution under UV–vis light irradiation (considering the solubility of H_3_BTC (26.3 mg·ml^−1^), always working under sink conditions). After 5 h, the sample was centrifuged, an aliquot of 100 μL was collected for HPLC analysis (At, SMT and H_3_BTC quantification), and the solid was resuspended again in a fresh At and SMT aqueous solution for 24 h. This process was repeated for 5 cycles.

## Supplementary Information

Supplementary Information.

## Data Availability

The datasets used and/or analysed during the current study available from the corresponding author on request.
