# Supplementary material for: Ti-based robust MOFs in the combined photocatalytic degradation of emerging organic contaminants
Source: Sci Rep. 2022 Aug 25;12:14513. doi: 10.1038/s41598-022-18590-1 (PMC9411604; doi:10.1038/s41598-022-18590-1)
Supplement: Supplementary file 1 — Supplementary Information. [file 41598_2022_18590_MOESM1_ESM.docx]

Supporting Information

**Sara Rojas,**^a,b,*^ **Jessica García-González,^c^ Pablo Salcedo-Abraira,**^a^ **Irene Rincón,**^a^ **Javier Castells-Gil,^d,e^ Natalia M. Padial,^d^ Carlos Marti-Gastaldo,^d^ Patricia Horcajada.^a,*^**

^a^ Advanced Porous Materials Unit (APMU), IMDEA Energy. Av. Ramón de la Sagra 3, 28935 Móstoles-Madrid, Spain.

^b^ Current address: Department of Inorganic Chemistry, University of Granada, Av. Fuentenueva S/N, 18071 Granada, Spain.

^c^ Department of Nursing, Physiotherapy and Medicine, Faculty of Health Sciences, University of Almería. 04120 Almería, Spain.

^d^ Instituto de Ciencia Molecular, Universitat de València, Catedrático José Beltrán, 2, 46980 Paterna, Spain.

^e^ Current address: School of Chemistry, University of Birmingham, Edgbaston, B15 2TT, Birmingham, United Kingdom.

**Table of contents**

S1. Ti-based MOFs screening…………………………………………………………………S2-9

S2. Identification of the photodegradation products…………………………….S10-S12

S3. Combined EOCs photodegradation………………………………………………….S13-S14

S4. Cyclability of MIL-100(Ti)…………………………………………………………………S15

S5. References………………………………………………………………………………………S16-S17

**S1. Ti-based MOFs screening**

**Table S1.** Brunauer, Emmett and Teller surface areas (S_BET_, m^2^·g^-1^) (p/p_0_ = 0.1 - 0.3), cavities and molecules dimensions (Å), pore window (Å) and volume (V_p_, cm^3^·g^-1^), and band gap (Eg, eV) for studied materials. External surface area was calculated by t-plot method (p/p_0_ from 0.3 to 0.6).

| **MOF** | **S_BET_**  **External Surface**  **(m^2^·g^-1^)** | **Dimensions (porosity or molecule size, Å)** | **Pore window**  **(Å)** | **V_p_**  **(cm^3^·g^-1^)** | **Eg (eV)**  Reported  Experimental | **Reported particle size**  **(µm)** |
| --- | --- | --- | --- | --- | --- | --- |
| MIL-100(Ti) | 1300  195 | 25 & 29 Å | 4.8x5.8 & 8.6 Å | 0.6 | 3.4^1^  3.48 | > 1 µm |
| MIL-100(Fe) | 1900  45.57 | 25 & 29 Å | 4.8x5.8 & 8.6 Å | 0.76 | 3.1^2^  2.73 | *ca.* 200 nm |
| MIL-125-NH_2_ | 1412  225 | 13 & 6 Å | 5-7 Å | 0.6 | 2.6^3^  2.53 | *ca.* 240 nm |
| MUV-10(Ca) | 1000  65 | 10 Å | 5 Å | 0.40 | 3.1^4^  3.63 | 50 µm |
| IEF-11 | 120  100 | 4.5 Å | 4.5 Å | 0.05 | 2.40^5^  2.40 | *ca.* 85 nm |
| At | - | 15 x 7 x 5 Å^3^ | - | - | - | - |
| SMT | - | 11 x 5 x 5 Å^3^ | - | - | - | - |

**Figure S1.** Band gap estimated from the Tauc plot for an indirect (or direct for IEF-11) allowed transition of MIL-100(Ti and Fe), MUV-10(Ca), MIL-125-NH2 and IEF-11.^6,7^

**Figure S2.** Photodegradation (triangles) and adsorption (squares) of At (black, left) over the time, as well as the ligand leaching (red, right) using different tested Ti-MOFs. The performance of MIL-100(Fe) has been added for comparison. For the IEF-11 leached linker study, it was measured after 5 h of experiment, achieving *ca.* 0.3% of MOF degradation.

**Figure S3.** Photodegradation (triangles) and adsorption (squares) of the SMT (black, left) as a function of the time, as well as the ligand leaching (red, right), using the different tested Ti-MOFs. The performance of MIL-100(Fe) has been added for comparison. For the IEF-11 leached linker study, it was measured after 5 h of experiment, achieving *ca.* 0.3% of MOF degradation.

**Figure S4.** Comparative evolution of At (a) and SMT (b) adsorption using different Ti-MOFs. For clarity, the degradation of MOFs is omitted here. Considering the pore size of IEF-11, smaller than the SMT and At dimensions, adsorption experiments were not performed using this material.

**Figure S5.** FTIR spectra of the original MIL-100(Ti), and its corresponding At loaded material (At@MIL-100(Ti)). The spectrum of free At has been included for comparison.

**Figure S6.** FTIR spectra of the original MIL-125-NH_2_, and its corresponding At (a, At@MIL-125-NH_2_) and SMT (b, SMT@MIL-125-NH_2_) loaded materials. The spectrum of free H_2_BDC-NH_2_ linker has been included for comparison.

**Figure S7.** X-ray powder diffraction (XRPD) patterns of the Ti-MOFs before (grey) and after been suspended in At tap-water solution either irradiated with UV-vis light (blue) or under dark (orange) after 24 h under stirring at room temperature (RT). XRPD pattern of MIL-100(Fe) has been added for comparison.

**Figure S8.** XRPD patterns of the Ti-MOFs before (grey) and after been suspended in SMT tap-water solution either irradiated with UV-vis light (blue) or under dark (orange) after 24 h under stirring at RT. XRPD pattern of MIL-100(Fe) has been added for comparison.

**Figure S9.** Fitting to a second order kinetics of At (a) and SMT (b) photodegradation data using MIL-125-NH_2_, MIL-100(Ti), MUV-10(Ca) and IEF-11.

**Table S2.** SMT and At catalytic degradation using other MOFs, MOF-based materials and other metallic species.

| **MOF** | **S_BET_**  **External Surface**  **(m^2^·g^-1^)** | **Dimensions (porosity or molecule size, Å)** | **Pore window**  **(Å)** | **V_p_**  **(cm^3^·g^-1^)** | **Eg (eV)**  Reported  Experimental | **Reported particle size**  **(µm)** |
| --- | --- | --- | --- | --- | --- | --- |
| MIL-100(Ti) | 1300  195 | 25 & 29 Å | 4.8x5.8 & 8.6 Å | 0.60 | 3.4^1^  3.48 | > 1 |
| MIL-100(Fe) | 1900  45 | 25 & 29 Å | 4.8x5.8 & 8.6 Å | 0.76 | 3.1^2^  2.73 | *ca.* 0.20 |
| MIL-125-NH_2_ | 1412  225 | 13 & 6 Å | 5-7 Å | 0.60 | 2.6^3^  2.53 | *ca.* 0.24 |
| MUV-10(Ca) | 1000  65 | 10 Å | 5 Å | 0.40 | 3.1^4^  3.63 | 50 |
| IEF-11 | 120  100 | 4.5 Å | 4.5 Å | 0.05 | 2.40^5^  2.40 | *ca.* 0.85 |
| At | - | 15 x 7 x 5 Å^3^ | - | - | - | - |
| SMT | - | 11 x 5 x 5 Å^3^ | - | - | - | - |

* In a mixture of contaminants (At and SMT).

**S2. Identification of the photodegradation products**

**Figure S10.** Potential SMT and At photodegradation positions.

**Figure S11.** MS spectra of the intermediate products detected upon the photocatalytic degradation of a) At (used as control), and b) and c) At in the presence of MIL-100(Ti) under UV-vis light irradiation.

**Figure S11.** MS spectra of the intermediates detected in the photocatalytic degradation of a) SMT (used as control), and b) and c) SMT in the presence of MIL-100(Ti) under UV-vis light irradiation.

**S3.** **Combined EOCs photodegradation**

**Figure S12.** XRPD patterns and FEG-SEM images of the original MIL-100(Ti) (black), and after the combined EOCs photodegradation (red) when using a) 35 and 5 ppm of At and SMT and 4 mg of MOF, respectively; b) half EOCs concentration: and c) half catalyst amount.

**Figure S13.** XRPD patterns of the original MIL-100(Ti) (black), and after the combined EOCs photodegradation (red) at initial pH a) 5.5 and b) 6.4.

**S4.** **Cyclability of MIL-100(Ti)**

**Figure S14.** XRPD patterns of the reported and original MIL-100(Ti) (black), and after 1, 2 and 5 cycles (red, blue and green, respectively) of the 5 h-photodegradation process of of At+SMT mixtures (total irradiation 25 h).

**S4. References**

1. Castells-Gil, J. *et al.* De novo synthesis of mesoporous photoactive titanium(IV)-organic frameworks with MIL-100 topology. *Chem. Sci.* **10**, 4313–4321 (2019).

2. Guesh, K. *et al.* Sustainable Preparation of MIL-100(Fe) and Its Photocatalytic Behavior in the Degradation of Methyl Orange in Water. *Cryst. Growth Des.* **17**, 1806–1813 (2017).

3. Hendon, C. H. *et al.* Engineering the Optical Response of the Titanium-MIL- 125 Metal-Organic Framework through Ligand Functionalisation. *J. Am. Chem. Soc.* 10942–10945 (2013).

4. Castells-Gil, J. *et al.* Chemical Engineering of Photoactivity in Heterometallic Titanium–Organic Frameworks by Metal Doping. *Angew. Chemie - Int. Ed.* **57**, 8453–8457 (2018).

5. Salcedo-Abraira, P. *et al.* A novel porous Ti-squarate as efficient photocatalyst in the overall water splitting reaction under simulated sunlight irradiation. *Adv. Mater.* **33**, 2106627 (2021).

6. Viezbicke, B. D., Patel, S., Davis, B. E. & Birnie, D. P. Evaluation of the Tauc method for optical absorption edge determination: ZnO thin films as a model system. *Phys. status solidi* **252**, 1700–1710 (2015).

7. SHIMADZU, n M. I. C. *Measurements of Band Gap in Compound Semiconductors - Band Gap Determination from Diffuse Reflectance Spectra*. https://www.shimadzu.com/an/sites/shimadzu.com.an/files/pim/pim_document_file/applications/application_note/14105/an_a428-en.pdf.

8. Li, R. *et al.* Improvement of Sulfamethazine photodegradation by Fe(III) assisted MIL-53(Fe)/percarbonate system. *Appl. Surf. Sci.* **457**, 726–734 (2018).

9. Li, G. *et al.* Solvent-free method to encapsulate polyoxometalate into metal-organic frameworks as efficient and recyclable photocatalyst for harmful sulfamethazine degrading in water. *Appl. Catal. B Environ.* **245**, 753–759 (2019).

10. Jia, M. *et al.* Integrating N and F co-doped TiO2 nanotubes with ZIF-8 as photoelectrode for enhanced photo-electrocatalytic degradation of sulfamethazine. *Chem. Eng. J.* **388**, 124388 (2020).

11. Tang, J. & Wang, J. Metal Organic Framework with Coordinatively Unsaturated Sites as Efficient Fenton-like Catalyst for Enhanced Degradation of Sulfamethazine. *Environ. Sci. Technol.* **52**, 5367–5377 (2018).

12. Yi, Z., Wang, J., Jiang, T., Tang, Q. & Cheng, Y. Photocatalytic degradation of sulfamethazine in aqueous solution using zno with different morphologies. *R. Soc. Open Sci.* **5**, (2018).

13. Guo, C. *et al.* Photodegradation of sulfamethazine in an aqueous solution by a bismuth molybdate photocatalyst. *Catal. Sci. Technol.* **3**, 1603–1611 (2013).

14. Zhou, C. *et al.* Visible-light-driven photocatalytic degradation of sulfamethazine by surface engineering of carbon nitride：Properties, degradation pathway and mechanisms. *J. Hazard. Mater.* **380**, 120815 (2019).

15. Mehrabadi, Z. & Faghihian, H. Comparative photocatalytic performance of TiO2 supported on clinoptilolite and TiO2/Salicylaldehyde-NH2-MIL-101(Cr) for degradation ofpharmaceutical pollutant atenolol under UV and visible irradiations. *J. Photochem. Photobiol. A* **356**, 102–111 (2018).

16. Ran, Z., Wang, L., Fang, Y., Ma, C. & Li, S. Photocatalytic degradation of atenolol by TIO2 irradiated with an ultraviolet light emitting diode: Performance, kinetics, and mechanism insights. *Catalysts* **9**, 1–17 (2019).
